# A first census of skin cancer specialist nurses across UK secondary care trusts

**DOI:** 10.1186/s12912-023-01374-x

**Published:** 2023-06-25

**Authors:** Jashmitha Rammanohar, Deeya Kotecha, Jackie Hodgetts, Saskia Reeken, Susanna Daniels, Pippa G Corrie

**Affiliations:** 1 University of Cambridge School of Clinical Medicine, Addenbrooke’s Hospital, Cambridge, England; 2 The Christie NHS Foundation Trust, Manchester, England; 3 Kingston Hospital NHS Foundation Trust, Kingston, England; 4 Melanoma Focus, Salisbury House, Station Road, Cambridge, England; 5 Department of Oncology, Cambridge University Hospitals NHS Foundation Trust, Cambridge, England

**Keywords:** Skin cancer, Melanoma, Specialist nurse, Secondary care, Resources, Workload, Lymphoedema, Telemedicine

## Abstract

**Background:**

Skin cancer specialist nurses (SCSNs) support patients and work alongside healthcare professionals throughout the care pathway. Skin cancer management is rapidly evolving, with increasing and more complex treatment options now available, so the need for patient support is growing. While SCSNs are a major source of that support, the provision of SCSN resource across the UK has never previously been assessed. We therefore undertook a first SCSN census on 1st June 2021.

**Methods:**

An electronic survey was disseminated to UK hospital trusts and registered skin cancer healthcare professionals. Responses were identifiable only by the respective trust name.

**Results:**

112 responses from 87 different secondary care trusts were received; 92% of trusts reporting having at least 1 established SCSN post. Average SCSN staffing per trust was 2.4 (range 0–7) whole time equivalents, managing an average caseload of 83 (range 6–400) patients per week. SCSN workload had increased in 82% hospitals in the previous year and 30% of trusts reported being under-resourced. Most SCSN time was spent managing melanoma (as opposed to non-melanoma skin cancer) patients linked to surgical services. Regional variations existed, particularly associated with provision of lymphoedema services, nurse prescribing skills and patient access to clinical trials. The COVID-19 pandemic was associated with a marked increase in SCSN-led telemedicine clinics, but loss of training and education opportunities.

**Conclusions:**

SCSNs based in secondary care hospitals play a major role supporting both clinicians and patients throughout the care pathway. This first UK census confirmed that SCSN workload is increasing and in one third of hospital trusts, the work was reported to outstrip the staffing available to manage the volume of work. Regional variations in SCSN resource, workload and job role, as well as availability of certain skin cancer services were identified, providing valuable information to healthcare commissioners concerned with service improvement.

**Supplementary Information:**

The online version contains supplementary material available at 10.1186/s12912-023-01374-x.

## Background

A cancer diagnosis is associated with considerable physical and psychosocial burden. Emotional support is important for most patients and their families [1]. The role of the treating team is considered pivotal to providing emotional support [2], as well as other aspects of practical and personalized support, helping with understanding of their disease and management [3]. Nurses as well as doctors and other allied healthcare professionals make up the treating team. In 2004, the National Institute for Health and Clinical Excellence (NICE) in England recommended that each cancer patient should have a ‘key worker’, responsible for supporting them, coordinating their care and being the point of contact for information and advice [4].

In the UK national health service (NHS), a range of types of specialist nurses work collaboratively in healthcare teams, providing an increasingly important contribution to the workforce, and most key workers are specialist nurses. The International Council of Nurses [5] distinguishes between specialised – as opposed to generalist – nurses, as well as advanced practice nurses who have gained a graduate degree to become a clinical nurse specialist (CNS), or nurse practitioner (NP). Specialist (ie. both specialised and advanced practice) nurses have acquired variable amounts of expert and specialist skills and knowledge, so they are capable of complex decision-making and able to adapt to contextual demand. Evidence demonstrates the beneficial role of specialist nurses extends well beyond improving patient wellbeing; they contribute to reducing the number of emergency hospital admissions, length of hospital stays and the volume of follow-up appointments [6].

Perhaps not surprisingly, specialist nurses are increasingly used as cost-effective surrogates for doctors and they can provide equal, or possibly, better care quality and outcomes compared to doctors in both primary [7] and secondary care settings [8]. This is particularly relevant to skin cancer, where treatment of melanoma has changed radically in the last 10 years: in addition to standard surgery to remove primary melanoma, many new and complex non-surgical treatments are now being offered to patients with metastatic melanoma and those at high risk of recurrence, which is keeping people alive much longer than ever before [9]. Similar innovations are occurring in the field of non-melanoma skin cancer, albeit of relevance to a smaller number of affected patients [10].

Skin cancer is the most common form of cancer in the UK, Europe and USA. Non-melanoma skin cancers – basal cell and squamous cell carcinoma – are the most common. The vast majority are readily diagnosed, removed by a small surgical procedure and have no lasting consequence for affected patients. Melanoma is less common, but is a far more aggressive form of skin cancer. Furthermore, melanoma incidence is rising annually; currently it is the 5th most common cancer in the UK, affecting both women and men and an incidence rise of 7% in the UK is predicted between 2014 and 2035 [11]. In the UK, skin cancer specialist nurses (SCSNs) based in secondary care hospital trusts are key workers for large numbers of patients with both melanoma and non-melanoma skin cancers. National melanoma patient management guidelines [12, 13] state that each local hospital skin cancer multidisciplinary team (MDT) and specialist skin cancer MDT should have at least one SCSN, who will play a leading role in supporting patients and their carers. NICE recommendations also state that all patients have the right to equity of access to information and support regardless of where the care is delivered. Despite national guidelines, anecdotally, access to SCSNs is known to vary widely across the country, but the actual numbers, workload and job roles of UK SCSNs is not known.

We undertook the first national SCSN census aimed at gathering information about the provision of SCSN posts across the UK, in order to investigate what level and type of support is being provided to patients diagnosed with and treated particularly for melanoma, according to NICE guidance, and assess any regional variations. This dataset would create a baseline for facilitating future skin cancer service development. As the census was undertaken during the first wave of the COVID-19 pandemic and a national ‘Lockdown’, we were also interested to understand what impact the pandemic had on SCSN clinical practice.

## Methods

This study was supported by the UK Melanoma Focus national charity and the British Association of Skin Cancer Specialist Nurses (BASCSN).

An electronic survey consisting of 24 questions assessing various aspects of the SCSN (including specialised and advanced practitioner) role was developed by a project management group, comprising a melanoma specialist medical oncologist (project lead) based at Cambridge University Hospitals NHS Foundation Trust (CUHFT), the Melanoma Focus Chief Executive Officer and CNS trustee member, a BASCSN NP and 2 Cambridge University medical students on their year 5 elective. The survey’s aim was to establish a baseline of SCSN resource, activities and workload in order to consider future service development needs. The key questions addressed were: what SCSN resource exists across UK hospitals? What work did they undertake? What was their caseload? Were there identifyable regional variations in resource provision? What was the impact of the first year of the COVID-19 pandemic on their work? What kind of job satisfaction did they have?

The survey was initially completed independently by 4 experienced SCSNs (1 NP, 1 CNS and 2 skin cancer specialised nurses working at CUHFT) who provided advice on the content before generating a final version. The electronic survey format was built by the medical students. A weblink to the electronic survey was sent to all secondary care trusts across the UK, disseminated by the East of England Cancer Alliance team. It was also shared with the BASCNS and Melanoma Focus professionals membership. The survey was disseminated during the week commencing 17th May 2021 and the Census date was 1st June 2021. Respondents were requested to complete the questionnaire once per hospital trust and the final date for submitting responses was 21st June 2021. All data was submitted anonymously, but respondents were asked to provide the name of their secondary care trust. The survey data was collated by the medical students and analysed by the project management group between 21st June and 27th August 2021. Trust responses were grouped geographically by their cancer alliance or devolved nation, in order to assess regional variations.

Where more than 1 response was received from a single trust, we took an average across the responses from that trust. Some survey questions required apportioning of time which should have totalled 100%. Where the totals were well out of range (> 120% or < 80%), the responses were omitted.

All methods were carried out in accordance with relevant guidelines and regulations. Advice was sought from the project lead’s institution (CUHFT) information and research governance lead, who confirmed that ethical approval and formal consent was not required to undertake this survey, which was completed voluntarily by anonymous individuals.

## Results

### Overview of skin cancer specialist nurse posts

**Fig. 1:**
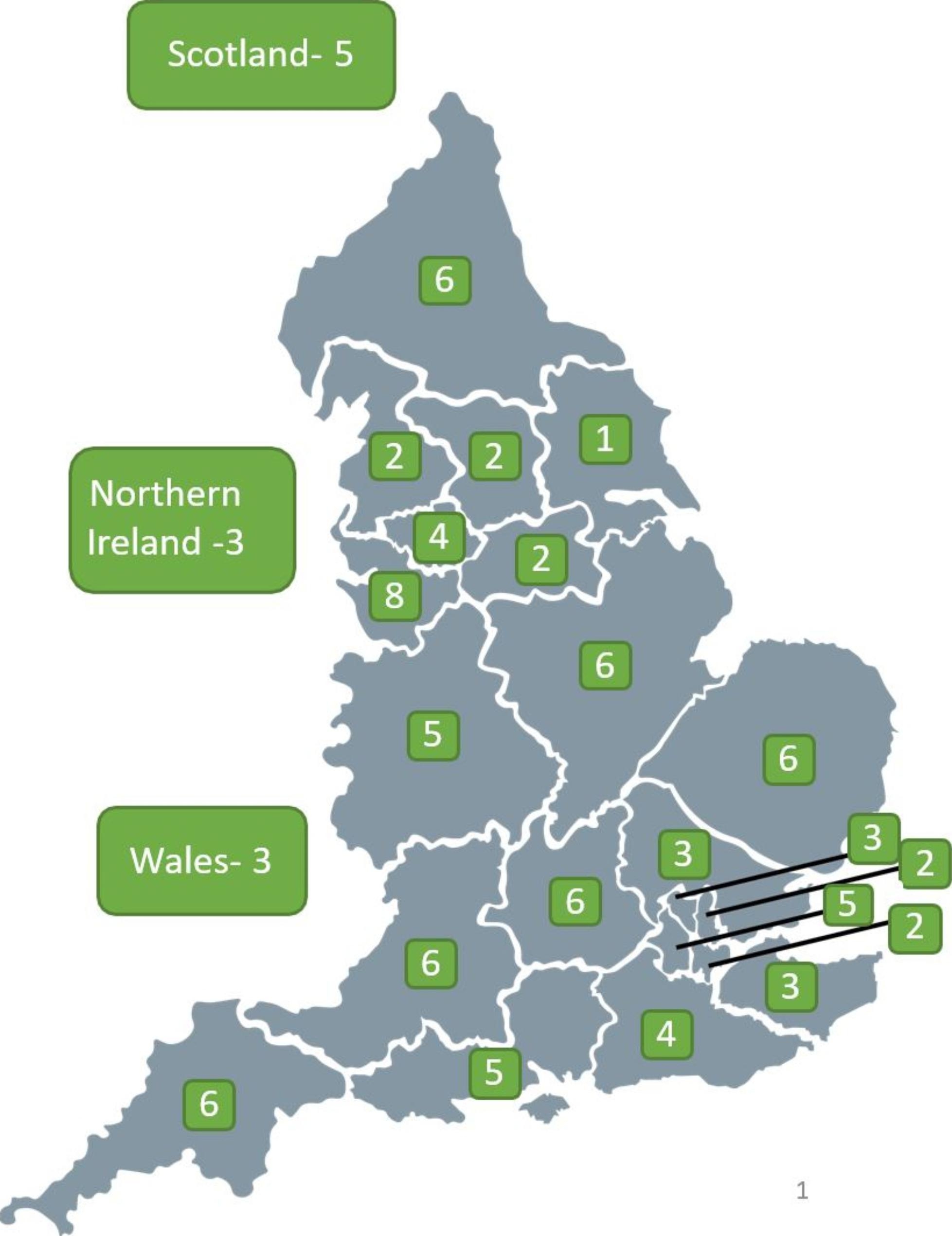
Map illustrating the number of different trusts that responded in each cancer alliance and devolved nation

A total of 112 survey responses were received, representing 87 different secondary care trusts in England, 5 trusts in Scotland, 3 trusts in Wales and 3 trusts in Northern Ireland. All 21 cancer alliances in England were represented. (Fig. 1). Four trusts reported not having a SCSN post, with no plans to create one, therefore 108 responses were subsequently analysed.

Overall, 92% of trusts confirmed they had at least 1 established SCSN post at the time of the Census (Table 1, Supplementary Fig. 1). Of those trusts without an established SCSN post, 56% stated there were plans to create a SCSN post.

**Table 1:** Summary of SCSN posts

Cancer Alliance / Devolved Nation	No. of trusts responding to survey	1 or more SCSN post established	WTE Mean	WTE Median	WTE Range	SCSN Posts Filled (% trusts)	AfC banding median (range)	Macmillan badged (% trusts)	Support worker (% trusts)
Cheshire and Merseyside	8	100%	2	1.5	0.8–6.4	88%	7 (6–8A)	88%	38%
East Midlands	6	100%	2.4	1.8	1.0–7.0	100%	7 (6–8B)	14%	43%
East of England – North	6	83%	1.9	2	0.6–3.0	100%	7 (4–7)	50%	33%
East of England – South	3*	100%	1.3	1.5	1.0–1.8	100%	7 (6–7)	75%	0%
Greater Manchester	4	100%	1.8	1.9	1.4–2.0	100%	7 (6–7)	50%	25%
Humber, Coast and Vale	1	100%	3	3	-	100%	7 (6–7)	0%	0%
Kent and Medway	3	67%	2.5	2.5	1.9–3.0	100%	7 (7–8A)	100%	50%
Lancashire and South Cumbria	2	100%	3.9	4	3.0–4.6	100%	7 (6–8A)	33%	33%
North Central London	3	75%	1.6	1.7	1.0–2.0	100%	7 (-)	0%	33%
North East London	2	67%	3.2	3	1.5–5.0	67%	7 (6–7)	33%	33%
Northern	6	75%	2.1	2	0–4.0	75%	7 (4–7)	88%	25%
Peninsula	6	100%	2.9	2.6	1.8–4.8	100%	7 (6–8A)	86%	50%
Royal Marsden Partners West London	5	100%	2.3	2.5	1.0–3.0	100%	8 (7–8B)	60%	40%
Somerset, Wiltshire, Avon and Gloucestershire	6	83%	2.3	2.2	1.6–4.0	100%	7 (6–8A)	50%	67%
South East London	2	100%	5.2	6	3.5–6.0	100%	7 (6–8A)	33%	67%
South Yorkshire and Bassetlaw	2	100%	2.2	2.2	1.3–3.0	100%	7 (6–7)	0%	0%
Surrey and Sussex	4	100%	1.9	2.1	1.0–2.8	67%	7 (4–8A)	100%	83%
Thames Valley	6	100%	1.3	1	1.0–1.8	67%	7 (6–8A)	100%	33%
Wessex	5	100%	1.9	2	0.8–3.0	83%	7 (4–8B)	17%	17%
West Midlands	5	83%	2.8	2	1.0–5.0	100%	7 (6–8A)	100%	0%
West Yorkshire and Harrogate	2	100%	2.4	2.4	1.8–3.0	50%	7 (6–7)	100%	50%
Northern Ireland	3	100%	2	1.7	1.0–3.7	100%	7 (5–8A)	100%	100%
Scotland	5	100%	1.6	1.7	1.0–2.0	100%	7 (6–7)	40%	20%
Wales	3	67%	2.1	2.1	1.0–3.2	100%	6 (6–7)	50%	0%
Overall	**98**	**92%**	**2.4**	**2.3**	**0–7.0**	**92%**	**7 (4–8B)**	**57%**	**35%**

*the respondent from 1 trust did not disclose their trust name so the true value may be 3 or 4; WTE: whole time equivalent; AfC: agenda for change pay scale (https://www.healthcareers.nhs.uk/working-health/working-nhs/nhs-pay-and-benefits/agenda-change-pay-rates/agenda-change-pay-rates)

The SCSN posts were predominantly based in dermatology departments within trusts (average 75%, range 0- 100), while 21/24 cancer alliances/devolved nations also had staff based in surgery and/or oncology departments. Twenty six (range 0–75)% of trusts had SCSN posts with distinct and separate primary remits (eg. supporting melanoma versus non-melanoma skin cancer patients, or early versus advanced melanoma patients); 55% (range 0–100%) of trusts stated that their SCSN posts provided cover within a wider team of specialist nurses.

### Skin cancer specialist nurse resource

The average SCSN resource across all cancer alliances/devolved nations was 2.4 whole time equivalents (WTE); ranging from 0–7 WTE across all trusts (Table 1, Supplementary Fig. 2). The total WTE identified in this census was 237. Most SCSN posts were filled at the time of the Census: 92% of trust posts were filled, with vacancies reported in 7 of 24 (29%) regions. The posts ranged from Agenda for Change (AfC, https://www.healthcareers.nhs.uk/working-health/working-nhs/nhs-pay-and-benefits/agenda-change-pay-rates/agenda-change-pay-rates) pay scale band 4 to 8B, with a median of band 7 in virtually all trusts. 57% of SCSN posts were supported to some extent by the Macmillan cancer charity, varying from 0–100% by cancer alliance/devolved nation; 35% (range 0–100%) of SCSN posts had a designated support worker.

### Management of different groups of skin cancer patients

**Fig. 2:**
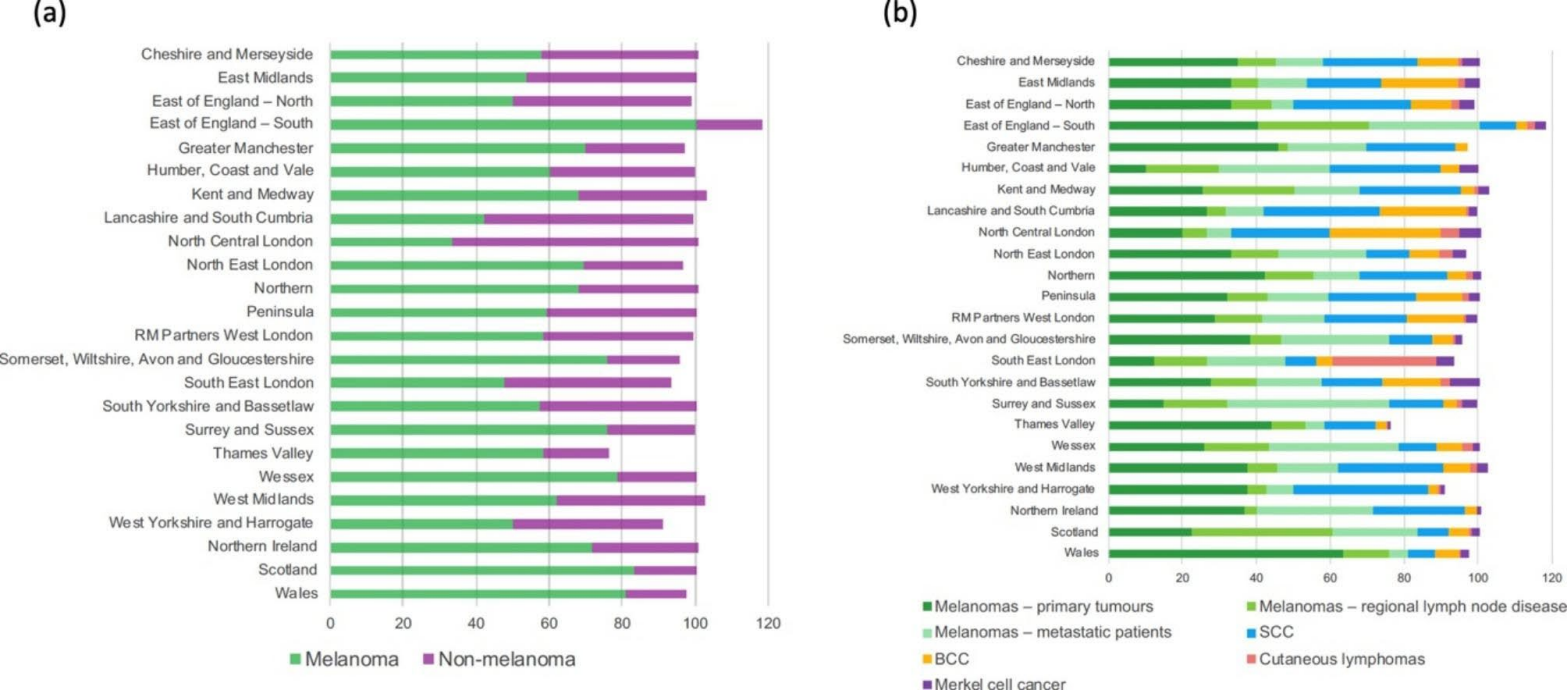
Proportion of SCSN time spent treating patients with (a) melanoma vs. non-melanoma skin cancer and (b) all skin cancer types including melanoma disease stages

The proportion of time spent by SCSNs managing patients with different types of skin cancer and melanoma disease stages by cancer alliance or devolved nation is shown in Fig. 2 and Supplementary Table 1. The split between melanoma and non-melanoma skin cancer work was 74 (range 33–85%)% versus 26 (range 7–68)% of time. Most time was spent on managing patients with primary disease, either melanoma 32 (range 10–64)%, or non-melanoma 26 (range 8–37)% skin cancer patients. Time spent managing loco-regional melanoma patients was 13 (range 3–38)% and metastatic melanoma patients was 19 (range 5–44)%.

### Skin cancer services

The survey asked what type of skin cancer services were available at the trust. Virtually all trusts (97%) provided surgical services for skin cancer (summarized in Table 2, with further details in Supplementary Table 2). Around half of trusts provided non-surgical oncological treatments (radiotherapy and systemic therapy). Two thirds of trusts offered photodynamic therapy (PDT), but only one third offered lymphoedema therapy. Clinical trial availability varied, but overall, 44% of trusts − 88% of regions - offered trials in melanoma and 35% of trusts − 75% of regions - in non-melanoma skin cancer (Supplementary Fig. 3).

The majority of SCSN time was spent providing support for patients undergoing surgery (60%, range 26–90%); 24% (range 0–49%) of time was spent with patients receiving systemic therapies and 8% (range 0–15%) radiotherapy. Only 2% (range 0–5%) of time was associated with skin cancer clinical trials (Supplementary Table 3, Supplementary Fig. 4).

**Table 2:** Percentage (%) of trusts overall and within each cancer alliance/devolved providing different types of skin cancer treatments and percentage (%) of SCSN time spent supporting patients receiving these treatments

	Surgery	Radio-therapy	Systemic therapy	Lymphoedema therapy	PDT	Melanoma clinical trials	Non-melanoma clinical trials
Overall % of trusts (range within regions)	97 (67–100)	56 0-100	53 0-100	38 0-100	65 0-100	44 0-100	35 0-100
Overall % of SCSN time (range within regions)	59 (26–90)	8 (0–17)	24 (0–49)	2 (0–7)	4 0–12	2 (0–5)	

PDT: photodynamic therapy

### Tasks undertaken by skin cancer specialist nurses

53% (range 0–100%) of SCSN time was reported to be spent working autonomously, independently of other healthcare professionals including doctors (Supplementary Table 4). Virtually all (99%) SCSNs undertook out-patient work, with slightly less (78%) undertaking in-patient work. There was a strong emphasis on liaison activity both with other secondary care trusts (undertaken by 96% of SCSNs) and the community support teams (undertaken by 92% of SCSNs), as opposed to direct management of patients in these settings (undertaken by 42% and 27% of SCSNs, respectively). SCSNs were working autonomously a lot of the time, able to independently assess patients (86% of SCSNs) and order various investigations (92% of SCSN). A minority (42%) of SCSNs were independent prescribers (Table 3, Supplementary Fig. 5).

**Table 3:** Type of work undertaken by SCSNs, reported by percentage (%) of trusts within each cancer alliance/devolved nation

Cancer Alliance / Devolved Nation	Outpatients (%)	Inpatients (%)	Managing patients in the community (%)	Liaison with community support teams (%)	Managing patients in other secondary care trust(s) (%)	Liaison with colleagues at other secondary care trust(s) (%)	Prescribing (%)	Independent assessment of patients (%)	Ordering of tests such as blood tests, scans (%)
Cheshire and Merseyside	88	38	13	63	63	100	75	100	88
East Midlands	100	71	14	100	43	100	14	86	86
East of England – North	100	80	20	100	60	100	40	100	100
East of England – South	100	100	50	100	100	100	0	75	100
Greater Manchester	75	75	25	100	0	100	75	100	100
Humber, Coast and Vale	100	100	0	100	0	100	0	100	100
Kent and Medway	100	100	0	100	50	100	0	100	100
Lancashire and South Cumbria	100	100	0	100	0	100	67	100	100
North Central London	100	100	0	100	0	100	0	67	100
North East London	100	50	0	50	0	100	50	50	100
Northern	100	100	67	100	83	100	33	83	83
Peninsula	100	100	17	83	33	100	50	100	100
RM Partners West London	100	100	20	100	0	100	60	100	80
Somerset, Wiltshire, Avon and Gloucestershire	100	80	40	80	60	100	20	100	100
South East London	100	100	67	100	67	100	100	100	100
South Yorkshire and Bassetlaw	100	50	0	100	50	50	50	100	100
Surrey and Sussex	100	100	50	100	50	100	83	100	83
Thames Valley	100	67	33	83	50	83	33	100	67
Wessex	100	83	17	83	50	83	50	33	83
West Midlands	100	80	20	100	60	100	40	60	80
West Yorkshire and Harrogate	100	50	50	100	50	100	0	50	100
Northern Ireland	100	50	25	75	25	100	100	75	75
Scotland	100	100	60	100	60	80	60	100	100
Wales	100	50	50	100	50	100	0	100	100
Overall	**99**	**79**	**27**	**92**	**42**	**96**	**42**	**86**	**93**

**Fig. 3:**
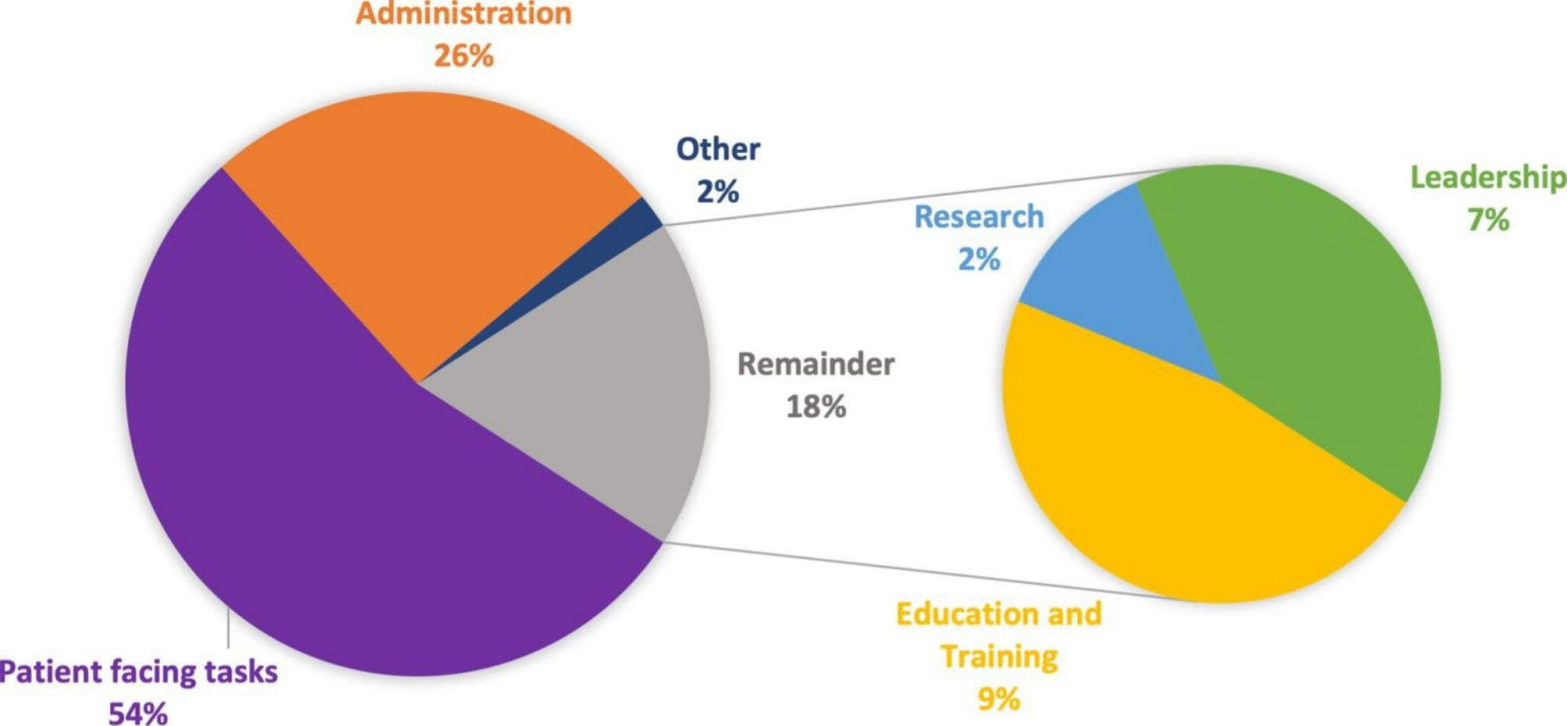
Proportion of SCSN time spent on different tasks during their working week

The proportion of time spent on patient-facing tasks (Fig. 3; Supplementary Table 5) was on average just over 50% (range 10–90%). The next highest category of time was spent on administration (average 26%, range 0–80%). On average, under 20% of time was spent on personal development: 8.7% (range 3.3–15%) on education and training, 7.5% (range 2.5–10%) on leadership tasks, with research featuring lowest on the priority list (average 2%, range 0–5%).

When working autonomously, on average, half of all SCSN time was spent seeing patients in follow-up/surveillance clinics (average 58%, range 0–100%), 13% (0–75%) in 2-week wait clinics and 21% (0-100) in other skin cancer specific clinics (Supplementary Table 6, Supplementary Fig. 6).

### Multidisciplinary (MDT) attendance

96% (range 75–100%) of SCSNs attended MDT meetings regularly (defined as every 1–2 weeks). 30% (0-100%) of SCSNs actually led the MDT meeting regularly, with a further 16% sometimes leading the MDT (Supplementary Table 7, Supplementary Fig. 7).

### Volume of work

The estimated case load in terms of number of patient contacts per week averaged 83 and ranged from 6 to 400 (Table 4, Supplementary Fig. 8). Trusts were asked to assess whether the case-load matched, exceeded or fell short of the WTE available to manage the work. 41% trusts reported the case load and SCSN WTE were matched. However, 32% of trusts reported the workload exceeded the SCSN WTE available. The overwhelming majority of trusts (82%) reported that their SCSN workload had increased over the previous year.

**Table 4:** Case load and comparison with SCSN WTE availability and workload change over the previous year

Cancer Alliance / Devolved Nation	Caseload	Matches WTE	Less than WTE	Exceeds WTE	Workload increased	Workload decreased	Workload stayed the same
Cheshire and Merseyside	110 (37–400)	25%	25%	50%	88%	12%	0%
East Midlands	98 (20–300)	57%	-	43%	86%	0%	14%
East of England – North	41 (10–80)	83%	-	17%	33%	17%	50%
East of England – South	31 (10–65)	50%	25%	25%	75%	25%	0%
Greater Manchester	48 (20–54)	25%	75%	-	75%	0%	25%
Humber, Coast and Vale	150	100%	-	-	100%	0%	0%
Kent and Medway	130 (110–150)	-	50%	50%	100%	0%	0%
Lancashire and South	131 (40–300)	33%	33%	33%	67%	0%	33%
North Central London	35 (30–40)	-	33%	67%	67%	0%	33%
North East London	92 (6-250)	33	-	67%	67%	0%	33%
Northern	77 (6-120)	38%	38%	25%	75%	0%	25%
Peninsula	99 30–210)	14%	71%	14%	86%	0%	14%
RM Partners West London	105 (20–300)	33	-	67%	100%	0%	0%
Somerset, Wiltshire, Avon and Gloucestershire	71 (30–190)	50%	33%	17%	100%	0%	0%
South East London	185 (40–216)	67%	-	33%	67%	0%	33%
South Yorkshire and Bassetlaw	90 (80–100)	50%	-	50%	100%	0%	0%
Surrey and Sussex	84 (20–150)	-	33%	67%	100%	0%	0%
Thames Valley	63 (20–100)	67%	33	-	83%	0%	17%
Wessex	55 (32–100)	50%	33%	1670%	67%	0%	33%
West Midlands	115 (25–200)	60%	40	-	80%	0%	20%
West Yorkshire and Harrogate	30	50%	50	-	100%	0%	0%
Northern Ireland	83 (60–120)	50%	25%	25%	100%	0%	0%
Scotland	59 (22–100)	40	-	60%	100%	0%	0%
Wales	42 (30–60)	50%	50	-	100%	0%	0%
Overall	**83**	**41%**	**27%**	**32%**	**82%**	**3%**	**15%**

WTE: whole time equivalent

### Impact of the COVID-19 pandemic on SCSN working

Trusts were asked to estimate the split between face-face and virtual/telephone contacts with patients across three time points: January 2020, January 2021 and January 2022 (Supplementary Table 8, Supplementary Fig. 9). Prior to the 2020 COVID-19 pandemic, face-face contact dominated in 22 of 24 (92%) regions, with an overall 2:1 ratio. In January 2021, in 14 of 24 (58%) regions, there was a shift away from face-face contacts in favour of telephone/virtual consultations, as measured by a minimum 10% change compared with the previous year. So, in January 2021, the overall ratio of contact method was 1:1. Interestingly, an overwhelming majority of trusts predicted a return to face-face contacts by January 2022, with an expectation to return to the 2020 2:1 ratio.

**Fig. 4:**
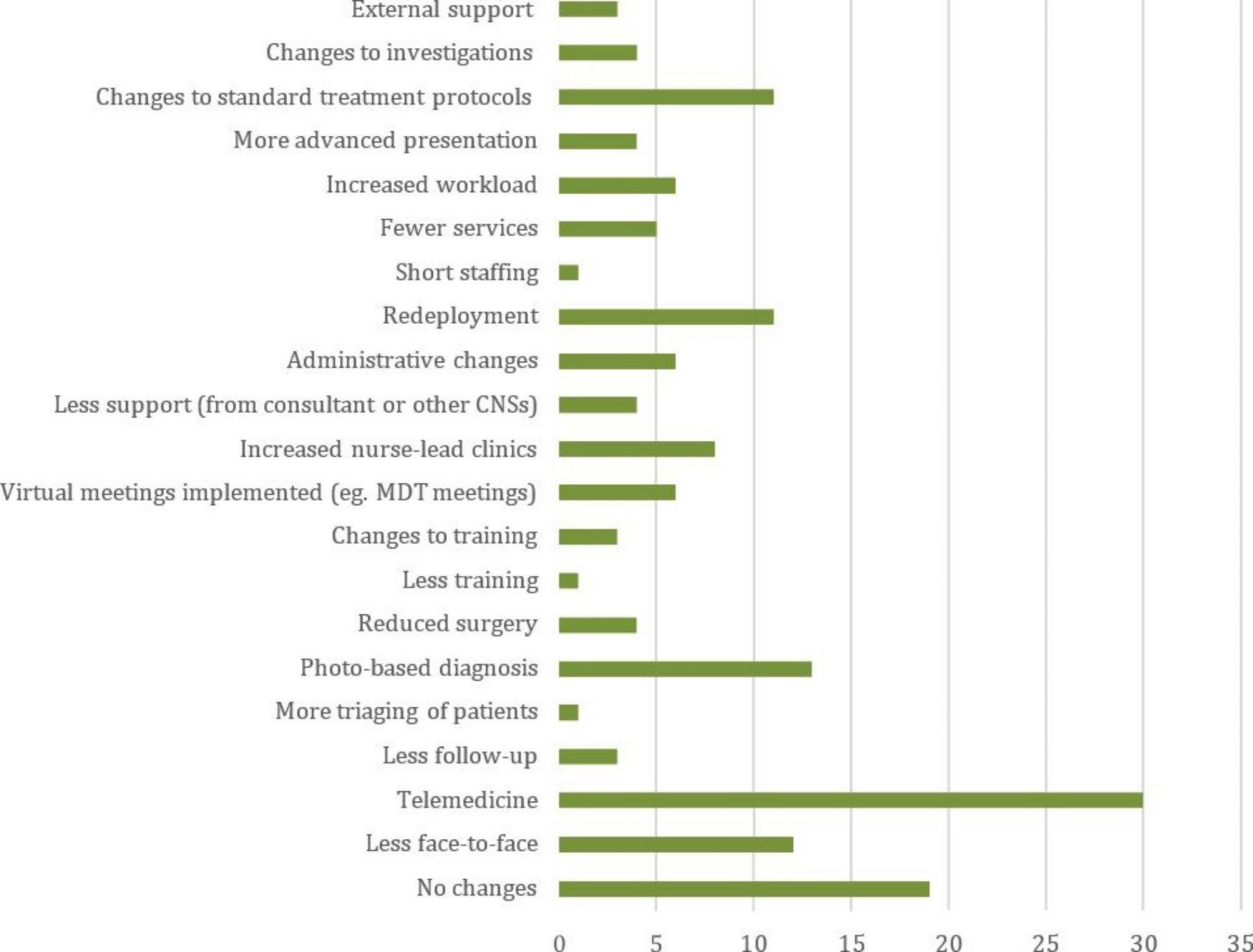
Thematic analysis of practice changes identified by respondents during the COVID-19 pandemic

Trusts were asked whether there were other key ways that practice had changed during the COVID-19 2020 pandemic. The key themes identified in Table 5 and summarized in Fig. 4. There was a major shift towards use of telemedicine in a variety of formats: remote telephone/virtual consultations, use of photography to assess pigmented and other lesions, as well as transfer of conducting MDT meetings and training from face-face to virtual platforms. Some positive interventions were mentioned such as the delivery of oral anti-cancer drugs to patients’ homes saving the need for patients to travel to hospitals to collect them. Other less positive experiences included a reduction in services available to patients during the pandemic, including surgery and specialist investigations, with concerns raised regarding the potential impact that patients might ultimately present with more advanced cancers. There was also a reduction in education and training opportunities for staff. Several comments related to an increase in workload with an emphasis on more nurse-led clinics, some with less consultant supervision. Some nurses were temporarily redeployed to support in-patient care for patients affected by COVID-19.

**Table 5:** Practice changes during the COVID-19 pandemic identified by respondents

Theme	Frequency	Relevant Examples
No changes	19	
Less face-to-face contact	12	“Most patients prefer face-face, and the telephone is not always suitable for all of the population i.e., deafness not aware why we were phoning” “We are short of space in the department due to social distancing with limited capacity in waiting area.” “We did move to virtual for patients but realized quickly that physical examination was important to us and patients as some patients presented later with new lesions”
Telemedicine	30	“Some patients preferred telephone but felt that (she) was missing clinical symptoms and progression” “Recognizing the limitations of virtual consultations” “Telephone monitoring clinics so relying on patients to notice skin changes.”
Less follow-up	3	
More triaging	1	
Photo-based diagnosis	13	
Delayed or reduced surgery	4	“No theatre capacity”
Less training	1	
Changes to training	3	
Virtual meetings	6	
Increased nurse-lead clinics	8	“If patients had to be seen then it was the SCSNs who undertook Face-face assessments”
Less support	4	
Administrative changes	6	“Tried to collate appointments to tie in with procedures, scans etc. to minimise (contact)” email to respond to patients’ queries
Redeployment	11	“Redeployed for 3 months to palliative COVID ward” “WTE of 3 due to redeployment”
Short staffing	1	
Fewer services	5	“Limited visits to inpatients” “Lack of CT scans, lack of SLNB, lack of MRI, lack of U/S.”
Increased workload	6	“Managing increased anxiety of patients with COVID” “More support calls as patients’ emotional needs are higher” “Had longer time between each patient due to the amount of cleaning to do” “Reliant on ad hoc clinics to match the number of referrals that are received.”
Patients’ presentation is more advanced	4	“Increased number of neglected tumours”
Changes to treatment protocols	11	“Treatment regime intervals changed and used more primary care support” “Home delivery of oral treatments”
Changes associated with investigations	4	“Community based blood tests.” “Histology and investigation results given by phone.”
External support	3	“Used more primary care support (GP), Linked more with other hospital (Pathology blood tests).” “Surgery carried out in the private sector”

### Job satisfaction

**Fig. 5:**
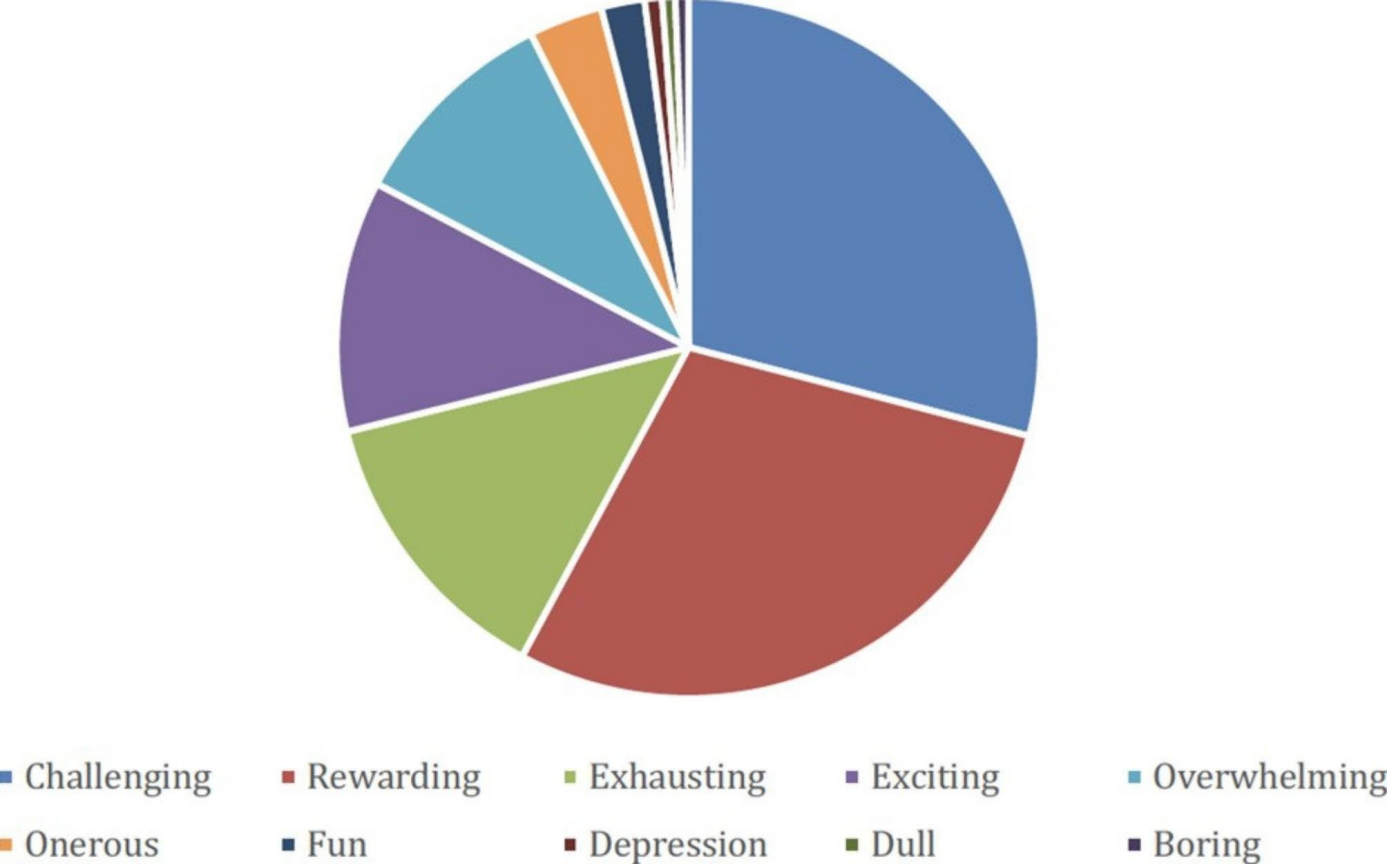
SCSN description of their job role

Finally, we asked colleagues responding with first-hand experience of undertaking the SCSN post to choose 3 words from a picklist of 10 words, which best described their job role (Fig. 5, Supplementary Table 9). A total of 354 selections were made. The most common descriptions of the SCSN post were: challenging (29%), and rewarding (29%), while the next most common descriptions were exhausting (13%), exciting (12%) and overwhelming (10%).

## Discussion

These data represent the first ever Census undertaken across the UK focusing specifically on SCSNs working in the NHS. Because of the uncertainty of the nature of the workforce, the Census did not define the term, ‘specialist nurse’, expecting that trusts could include data on any nurses working within the skin cancer healthcare team and thereby having some degree of specialist knowledge and/or expertise in managing skin cancer patients. This might therefore include both specialised and advanced practice (CNS and NP) nurses. The survey was completed anonymously, so we have limited information regarding who actually submitted the data in each trust and where the data was sourced from. However, from communications received following circulation of the survey, our impression is that most of the data was submitted by individuals currently working in SCSN posts. Based upon information supplied in the Macmillan specialist nurse census undertaken in 2017 [14], there are 149 secondary care trusts across England caring for adult cancer patients (excluding paediatric hospitals and very specialist trusts). We received data from 87 trusts in England, which therefore represents 58% of all trusts. However, we don’t know how many of all secondary care trusts offer dermatology services – some may not. Responses from trusts in the devolved nations were limited and we do not have comparator figures for the number of trusts in these countries. Even so, the volume of responses was, in our view, encouraging and evidence of a motivated and engaged skin cancer health care professional community. Their engagement suggests that the data is likely to be a fair representation of work undertaken by SCSNs across the country.

### Skin cancer specialist nurse resource

We have compared our findings to data reported by the 2017 Macmillan Census [14], which covered a broad range of cancer, palliative and specialist nurses. The Macmillan Census provides some statistics on specialist nurses in general as well as those working with skin cancer patients, designated ‘malignant dermatology’. The Macmillan Census identified a total of 204 WTEs of specialist nurse time working in malignant dermatology, representing 5% of the cancer site-specific specialist nurse workforce in England. Our 2021 census identified a total of 237 WTEs of SCSNs existing in what we suspect to be less than two thirds of UK trusts. Scaled up, this would suggest a significant expansion in the SCSN workforce in the last 4 years and this could well reflect both the increase in number of skin cancer cases alongside a significant increase in treatment options for these patients requiring more service support.

In 2017, Macmillan noted that 66% of malignant dermatology specialist nurse posts were AfC band 7, ranging from band 6–8D and 42% were Macmillan-badged. In 2021, similarly, the median banding of SCSNs was 7, but the range of post grades was trending downwards, from 4–8B. AfC is the national pay system for all NHS staff with the exception of doctors, dentists and senior managers. A set of 9 pay bands are based on the employee role and responsibilities for which a set of national job profiles were defined to assist in matching jobs and pay across different health and care organisations. Examples of matched nursing job profiles are: staff nurse = band 5, specialist staff nurse or junior sister = band 6, advanced nurse practitioner or senior sister = band 7 and senior nurse manager/matron = band 8. Given the wide variation in AfC banding allocated to SCSNs, it is clear that the expectations of their responsibilities must differ significantly in different hospitals and reflects a blurring of definition of the specialist nurse role. A good example is that a band 4 post is more likely to be a skin cancer support worker, not a qualified nurse, but someone working under the management of the specialist nurse team and still providing a huge contribution to patients and their professional team. Overall, we observed a downward trend of specialist nurse post banding over time, which was also identified in the 2017 Macmillan Census, comparing data collected in 2014. This and the higher rate of 57% posts being Macmillan-badged in 2021, may well reflect increasing financial pressures within the NHS and reliance on external funding sources to facilitate NHS activity. The trend towards lower pay-band posts is concerning given the high levels of responsibility reported to be undertaken by the SCSNs during their working week in this survey: 53% of specialist nurse time was spent working autonomously, with tasks including independent assessment of patients (86%), ordering investigations (92%), prescribing drugs (42%) and leading MDT meetings (16%). A further concern was that overall time spent on personal development (education, training, research and leadership totaling 18.5%) was less than that spent on routine administrative tasks (26%).

### Caseload and impact of the COVID-19 pandemic

Using a 2015 malignant skin cancer incidence of 13,356 cases for England, Macmillan estimated the ratio of new patients to malignant dermatology specialist nurse (calculated based on WTE) to be 65. Taking the more recent CRUK 2016-18 average melanoma incidence figure of 16,175 [11] for the whole of the UK, the ratio of new patients to SCSN in our 2021 Census is 68. While not dissimilar to the Macmillan figure, the caveats are that our Census data is both limited and unverified. On the other hand, from the data we collected from trusts regarding individual nurse caseload, the Census suggests a higher volume of work, with an average of 83 patient contacts per week, albeit with significant variations across trusts and regions.

A clear message is that the SCSN overall workload is increasing and in many trusts, the work outstrips the staffing available to manage the volume of patients. COVID-19 has added to the challenges that all healthcare professionals face and while positive experiences linked to modern technology and telemedicine were reported, there were concerns raised that interruption of standard diagnostic and treatment pathways may have been detrimental to patients and their disease outcomes. Further work to measure these changes is needed to mitigate against similar risks in future pandemics.

Given the lack of known denominators, it is difficult to draw any firm conclusions regarding specific geographical variations in SCSN provision. However, we did observe a lack of lymphoedema services in some regions, which is an important element of skin cancer care provision. Furthermore, not all regions had access to skin cancer clinical trials, suggesting inequity of service quality across the country, since research drives better patient outcomes.

### Job satisfaction

The SCSN perceptions of their job role illustrate well the good and bad aspects of working in a busy, rapidly evolving specialist area of modern cancer medicine. The picklist of descriptions offered was not a validated tool, but the array of choices demonstrate the positive descriptions of ***Rewarding ****and ****Exciting*** are counterbalanced with those of ***Challenging, Exhausting*** and, indeed, ***Overwhelming***. Given that most trusts reported increased workload over the last year that is outstripping the available SCSN support in many regions, there is a clear message here that additional resource is needed to build an appropriate workforce needed to provide an optimal service for our skin cancer patients.

### Study limitations

This Census clearly has several limitations. This survey was not conducted with the rigors of formal qualitative research. While we have still learned a lot about the work undertaken by SCSNs in those trusts who responded, we do not know the situation in those trusts who did not respond. We are making a presumption that the data collected is representative of the national as a whole. Because of the anonymity, we were not able to verify or validate the information supplied. So, for example, we were surprised to see 1 trust report 7 SCSN WTEs, which is well above the average 2.3 WTEs. It is possible that the individual completing the survey mistook ‘WTE’ for number of nurses in post, or it may be true evidence of the wide variation in resource availability between trusts. There was also a large variation in patient caseload reported, but the number of cases were estimated and verification from a reliable source was not required. These aspects could be addressed in a future Census and other ways of seeking a more complete dataset should be considered.

## Conclusions

From this first UK SCSN census, we conclude with the following recommendations:

1. The Census has identified a SCSN workforce, which is growing to meet the increase in demand, likely driven by rising skin cancer incidence, increasing treatment options for, in particular, melanoma patients, and the consequential increase in survivorship. Even so, one third of regions appear to be under-resourced and action is needed to address staffing requirements in these respective cancer alliances and devolved nations.
2. We have identified a potential lack of lymphoedema services in some regions. Given the impact of lymphoedema on patient quality of life, a national priority should be to ensure that all patients can access specialist support equally, wherever they are living.
3. All regional research networks should review their provision of skin cancer clinical trials and ensure at least 1 specialist centre in their region provides patients with access to clinical research opportunities in both melanoma and non-melanoma skin cancer.
4. Trusts should review the AfC pay-scales of their SCSNs to ensure that their roles and responsibilities are fairly matched. Furthermore, adequate time should be protected in their job plans to ensure personal development is prioritized.
5. The impact of the COVID-19 pandemic on SCSN working is noteworthy, with many negative themes identified. Safe-guarding the wellbeing of all staff as the country emerges from the pandemic is clearly a priority for all NHS managers and our recommendation is to continue to work closely with individual staff groups who will be affected in different ways. Although the expansion of telemedicine has its values, the SCSN community shared a strong hope to return to more face-face patient contact, likely perceiving benefits for patients as well as to their own enjoyment of work. Trusts need to evaluate the growing use of telemedicine to ensure that its use is proportionate to need.
6. This 2021 Census if the first of its kind undertaken specifically to focus on the SCSN workforce. It builds on previous data generated by Macmillan in 2017, but provides only a snapshot of activity in a proportion of UK trusts. Methods to ensure better national coverage should be incorporated into future versions, potentially with mechanisms to ensure key data can be verified.

## Electronic supplementary material

Below is the link to the electronic supplementary material.

Supplementary Material 1

## Data Availability

A link to the Census e-Survey can be found at: https://docs.google.com/forms/d/1JLXFZiXwth1wteC5Q8Uayd0YZA4RZq6KGnLzNoSgSrM/edit?usp=sharing. Detailed data from the survey is contained in the Additional Information File accompanying the main manuscript.

## Abbreviations

AfC: Agenda for change
BASCSN: British Association of Skin Cancer Specialist Nurses
CNS: Clinical nurse specialist
CUHFT: Cambridge University Hospitals NHS Foundation Trust
MDT: Multidisciplinary team
MF: Melanoma Focus
NHS: National Health Service
NICE: National Institute for Health and Clinical Excellence
NP: Nurse practitioner
PDT: Photodynamic therapy
SCSN: Skin cancer specialist nurse
WTE: Whole time equivalent
