# Supplementary material for: A first census of skin cancer specialist nurses across UK secondary care trusts
Source: BMC Nurs. 2023 Jun 25;22:216. doi: 10.1186/s12912-023-01374-x (PMC10290789; doi:10.1186/s12912-023-01374-x)

**A first census of skin cancer specialist nurses across UK secondary care trusts**

**Supplementary Information**

**Supplementary Table 1: Proportion of time spent by SCSNs treating different types of skin cancer and melanoma disease stages by cancer alliance/devolved nation**

| **Cancer Alliance / Devolved Nation** | **Number of responses** | | **Melanoma - primary tumours (%)** | **Melanoma - regional lymph node disease (%)** | **Melanoma - metastatic (%)** | **SCC (%)** | **BCC (%)** | **Cutaneous lymphoma (%)** | **Merkel cell cancer (%)** |
| --- | --- | --- | --- | --- | --- | --- | --- | --- | --- |
| **Cheshire and Merseyside** | 8 | | 35 | 10 | 13 | 26 | 11 | 1 | 5 |
| **East Midlands** | 6* | | 33 | 7 | 13 | 20 | 21 | 2 | 4 |
| **East of England – North** | 6 | | 33 | 11 | 6 | 32 | 11 | 2 | 4 |
| **East of England – South** | 4 | | 41 | 30 | 30 | 10 | 3 | 2 | 3 |
| **Greater Manchester** | 4 | | 46 | 3 | 21 | 24 | 3 | 0 | 0 |
| **Humber, Coast and Vale** | 1 | | 10 | 20 | 30 | 30 | 5 | 0 | 5 |
| **Kent and Medway** | 2 | | 26 | 25 | 18 | 28 | 4 | 1 | 3 |
| **Lancashire and South** | 3 | | 27 | 5 | 10 | 31 | 23 | 1 | 2 |
| **North Central London** | 3 | | 20 | 7 | 7 | 27 | 30 | 5 | 6 |
| **North East London** | 3 | | 33 | 13 | 24 | 12 | 8 | 4 | 4 |
| **Northern** | 7* | | 42 | 14 | 12 | 21 | 5 | 2 | 2 |
| **Peninsula** | 6* | | 32 | 11 | 16 | 24 | 12 | 2 | 3 |
| **RM Partners West London** | 5 | | 29 | 13 | 17 | 22 | 16 | 1 | 3 |
| **Somerset, Wiltshire, Avon and Gloucestershire** | 6 | | 38 | 8 | 29 | 12 | 6 | 1 | 2 |
| **South East London** | 3 | | 13 | 14 | 21 | 8 | 4 | 28 | 2 |
| **South Yorkshire and Bassetlaw** | 2 | | 28 | 13 | 18 | 17 | 16 | 3 | 8 |
| **Surrey and Sussex** | 6 | | 15 | 17 | 44 | 14 | 4 | 2 | 4 |
| **Thames Valley** | 5 | | 44 | 9 | 5 | 14 | 3 | 0 | 1 |
| **Wessex** | 6 | | 26 | 18 | 35 | 10 | 7 | 3 | 2 |
| **West Midlands** | 4* | | 38 | 8.3 | 16 | 29 | 7 | 2 | 3 |
| **West Yorkshire and Harrogate** | 2 | | 38 | 5 | 8 | 37 | 3 | 1 | 1 |
| **Northern Ireland** | 3* | 37 | | 3 | 32 | 25 | 3 | 0 | 1 |
| **Scotland** | 4* | 23 | | 38 | 23 | 9 | 6 | 1 | 2 |
| **Wales** | 2 | 64 | | 13 | 5 | 8 | 7 | 1 | 2 |
| **Overall** | **101** | **32** | | **13** | **19** | **20** | **9** | **3** | **3** |

*This is the actual data provided by respondents, not all total 100%. 7 responses were excluded because 6 trusts submitted data which totaled either <80% or >120% and 1 response was unsure of values.

**Supplementary Table 2: Type of skin cancer treatment available, by proportion of trusts within each cancer alliance/devolved nation**

| **Cancer Alliance / Devolved Nation** | **Surgery (%)** | **Radiotherapy (%)** | **Systemic therapy (%)** | **Lymphoedema therapy (%)** | **Photodynamic therapy (%)** | **Clinical trials specifically for melanoma patients (%)** | **Clinical trials specifically for non-melanoma cancer patients** |
| --- | --- | --- | --- | --- | --- | --- | --- |
| **Cheshire and Merseyside** | 88 | 25 | 25 | 13 | 50 | 25 | 38 |
| **East Midlands** | 100 | 57 | 57 | 29 | 71 | 29 | 14 |
| **East of England – North** | 100 | 50 | 33 | 33 | 50 | 33 | 33 |
| **East of England – South** | 100 | 50 | 50 | 75 | 100 | 25 | 25 |
| **Greater Manchester** | 100 | 25 | 25 | 50 | 0 | 50 | 50 |
| **Humber, Coast and Vale** | 100 | 100 | 100 | 100 | 0 | 100 | 100 |
| **Kent and Medway** | 100 | 50 | 50 | 0 | 100 | 0 | 10 |
| **Lancashire and South** | 100 | 67 | 33 | 0 | 67 | 67 | 33 |
| **North Central London** | 100 | 100 | 50 | 0 | 50 | 50 | 50 |
| **North East London** | 100 | 100 | 67 | 67 | 67 | 33 | 67 |
| **Northern** | 100 | 38 | 38 | 38 | 75 | 38 | 38 |
| **Peninsula** | 100 | 86 | 100 | 71 | 86 | 71 | 86 |
| **RM Partners West London** | 100 | 40 | 60 | 20 | 80 | 60 | 20 |
| **Somerset, Wiltshire, Avon and Gloucestershire** | 100 | 50 | 50 | 50 | 67 | 33 | 50 |
| **South East London** | 67 | 67 | 100 | 100 | 100 | 100 | 67 |
| **South Yorkshire and Bassetlaw** | 100 | 50 | 50 | 0 | 50 | 50 | 0 |
| **Surrey and Sussex** | 100 | 83 | 67 | 50 | 67 | 50 | 33 |
| **Thames Valley** | 100 | 33 | 50 | 33 | 67 | 33 | 33 |
| **Wessex** | 100 | 83 | 67 | 67 | 83 | 50 | 33 |
| **West Midlands** | 100 | 40 | 40 | 20 | 100 | 20 | 0 |
| **West Yorkshire and Harrogate** | 100 | 0 | 0 | 0 | 100 | 0 | 0 |
| **Northern Ireland** | 100 | 50 | 50 | 0 | 25 | 50 | 0 |
| **Scotland** | 80 | 100 | 100 | 40 | 60 | 100 | 60 |
| **Wales** | 100 | 0 | 0 | 50 | 50 | 0 | 0 |
| **Overall** | **97** | **56** | **53** | **38** | **65** | **44** | **35** |

**Supplementary Table 3: Proportion of time spent by SCSNs providing support for patients receiving different skin cancer treatments**

| **Cancer Alliance / Devolved Nation** | **Number of trusts** | **Surgery (%)** | **Radiotherapy (%)** | **Systemic therapy (%)** | **Lymphoedema therapy (%)** | **Photodynamic therapy (%)** | **Skin cancer clinical trial (%)** |
| --- | --- | --- | --- | --- | --- | --- | --- |
| **Cheshire and Merseyside** | 8 | 71 | 2 | 18 | 1 | 2 | 4 |
| **East Midlands** | 7 | 66 | 6 | 1 | 1 | 9 | 1 |
| **East of England – North** | 4 | 73 | 11 | 8 | 3 | 5 | 3 |
| **East of England – South** | 4 | 55 | 3 | 38 | 2 | 2 | 1 |
| **Greater Manchester** | 4 | 68 | 4 | 21 | 3 | 0 | 4 |
| **Humber, Coast and Vale** | 1 | 30 | 15 | 40 | 5 | 5 | 5 |
| **Kent and Medway** | 2 | 83 | 5 | 10 | 0 | 3 | 0 |
| **Lancashire and South Cumbria** | 3 | 67 | 8 | 13 | 0 | 8 | 3 |
| **North Central London** | 3 | 73 | 12 | 5 | 0 | 3 | 4 |
| **North East London** | 2 | 38 | 17 | 23 | 3 | 9 | 1 |
| **Northern** | 7 | 73 | 6 | 14 | 4 | 2 | 1 |
| **Peninsula** | 3 | 60 | 3 | 32 | 0 | 3 | 2 |
| **RM Partners West London** | 6 | 50 | 13 | 30 | 0 | 2 | 3 |
| **Somerset, Wiltshire, Avon and Gloucestershire** | 5 | 59 | 8 | 25 | 0 | 7 | 1 |
| **South East London** | 4 | 38 | 10 | 41 | 4 | 4 | 5 |
| **South Yorkshire and Bassetlaw** | 6 | 57 | 6 | 33 | 3 | 2 | 1 |
| **Surrey and Sussex** | 3 | 26 | 11 | 42 | 7 | 12 | 0 |
| **Thames Valley** | 1 | 45 | 10 | 45 | 0 | 0 | 0 |
| **Wessex** | 6 | 35 | 13 | 49 | 2 | 1 | 3 |
| **West Midlands** | 5 | 68 | 8 | 17 | 3 | 2 | 2 |
| **West Yorkshire and Harrogate** | 2 | 90 | 3 | 3 | 3 | 2 | 1 |
| **Northern Ireland** | 6 | 44 | 14 | 33 | 3 | 3 | 1 |
| **Scotland** | 5 | 68 | 8 | 19 | 3 | 2 | 1 |
| **Wales** | 2 | 90 | 0 | 0 | 0 | 0 | 0 |
| **Overall** | **99** | **59** | **8** | **24** | **2** | **4** | **2** |

These are the actual data provided by respondents, not all total 100%. 9 responses were excluded because 8 trusts submitted data which totaled either <80% or >120% and were 1 response was unsure of value.

**Supplementary Table 4: Proportion of time the SCSNs were reported to work autonomously**

| **Cancer Alliance / Devolved Nation** | **Time spent working autonomously**  **Average (range)** |
| --- | --- |
| **Cheshire and Merseyside** | 67 (50-80)% |
| **East Midlands** | 64 (20-100)% |
| **East of England – North** | 38 (11-80)% |
| **East of England – South** | 44 (0-80)% |
| **Greater Manchester** | 69 (0-100)% |
| **Humber, Coast and Vale** | 50% |
| **Kent and Medway** | 55 (40-70)% |
| **Lancashire and South Cumbria** | 73 (60-90)% |
| **North Central London** | 32 (10-60)% |
| **North East London** | 60% |
| **Northern** | 72 (10-100)% |
| **Peninsula** | 39 (10-80)% |
| **RM Partners West London** | 70 (50-80)% |
| **Somerset, Wiltshire, Avon and Gloucestershire** | 44 (25-80)% |
| **South East London** | 48 (0-70)% |
| **South Yorkshire and Bassetlaw** | 70 (50-90)% |
| **Surrey and Sussex** | 47 (0-80)% |
| **Thames Valley** | 57 (50-80)% |
| **Wessex** | 36 (0-100)% |
| **West Midlands** | 51 (21-70)% |
| **West Yorkshire and Harrogate** | 50% |
| **Northern Ireland** | 53 (50-62.5)% |
| **Scotland** | 46 (20-100)% |
| **Wales** | 48 (20-75)% |
| **Overall** | **53 (0-100)%** |

**Supplementary Table 5: Proportion of SCSN time spent on different tasks**

| **Cancer Alliance / Devolved Nation** | **Patient facing tasks (%)** | **Administration (%)** | **Education and training (%)** | **Research (%)** | **Leadership (%)** | **Other (%)** |
| --- | --- | --- | --- | --- | --- | --- |
| **Cheshire and Merseyside** | 68 | 18 | 6.3 | 0.8 | 4.8 | 0 |
| **East Midlands** | 62 | 27 | 6.4 | 0.7 | 7.1 | 0 |
| **East of England – North** | 54 | 31 | 5.8 | 2.8 | 4.7 | 1.7 |
| **East of England – South** | 42 | 45 | 7.5 | 0 | 2.5 | 0 |
| **Greater Manchester** | 70 | 11 | 6.3 | 2.5 | 7.5 | 2.5 |
| **Humber, Coast and Vale** | 45 | 25 | 10 | 10 | 10 | 0 |
| **Kent and Medway** | 62 | 18 | 10 | 2.5 | 5 | 2.5 |
| **Lancashire and South** | 75 | 13 | 3.3 | 0 | 1.7 | 6.7 |
| **North Central London** | 40 | 27 | 15 | 3.7 | 13 | 3.3 |
| **North East London** | 31 | 39 | 11 | 5 | 5 | 8.3 |
| **Northern** | 61 | 24 | 7.5 | 1.7 | 5.7 | 0.7 |
| **Peninsula** | 56 | 22 | 7.6 | 3.1 | 11 | 0 |
| **RM Partners West London** | 64 | 16 | 11 | 2 | 7 | 1 |
| **Somerset, Wiltshire, Avon and Gloucestershire** | 53 | 24 | 9 | 3.5 | 10 | 3.3 |
| **South East London** | 49 | 23 | 15 | 1.7 | 10 | 3.3 |
| **South Yorkshire and Bassetlaw** | 60 | 30 | 7.5 | 0 | 2.5 | 0 |
| **Surrey and Sussex** | 58 | 28 | 5.8 | 1.7 | 6.7 | 0.8 |
| **Thames Valley** | 52 | 27 | 8 | 0.4 | 7 | 5.6 |
| **Wessex** | 46 | 38 | 4.2 | 2 | 5.5 | 1.7 |
| **West Midlands** | 54 | 26 | 9 | 2 | 6 | 3 |
| **West Yorkshire and Harrogate** | 55 | 20 | 15 | 0 | 30 | 0 |
| **Northern Ireland** | 61 | 22 | 7 | 2 | 5.8 | 2.5 |
| **Scotland** | 59 | 19 | 8.8 | 3.8 | 10 | 0 |
| **Wales** | 30 | 50 | 15 | 5 | 2.5 | 2.5 |
| **Overall** | **55** | **26** | **8.7** | **2.3** | **7.5** | **2** |

**Supplementary Table 6: Proportion of time spent by SCSNs working autonomously in different types of clinics**

| **Cancer Alliance / Devolved Nation** | **2 week wait clinic (%)** | **Follow-up / surveillance clinic (%)** | **Other skin cancer specific clinic (%)** | **Other (%)** |
| --- | --- | --- | --- | --- |
| **Cheshire and Merseyside** | 16 | 61 | 14 | 8.9 |
| **East Midlands** | 21 | 48 | 14 | 4.4 |
| **East of England – North** | 4.2 | 73 | 19 | 3.3 |
| **East of England – South** | 16 | 67 | 17 | 0 |
| **Greater Manchester** | 2.5 | 71 | 11 | 14 |
| **Humber, Coast and Vale** | 0 | 100 | 0 | 0 |
| **Kent and Medway** | 2.5 | 80 | 18 | 0 |
| **Lancashire and South Cumbria** | 27 | 63 | 10 | 0 |
| **North Central London** | 42 | 27 | 27 | 5 |
| **North East London** | 20 | 27 | 6.7 | 37 |
| **Northern** | 1.3 | 60 | 8.8 | 6.3 |
| **Peninsula** | 2 | 64 | 31 | 3 |
| **RM Partners West London** | 9 | 46 | 34 | 16 |
| **Somerset, Wiltshire, Avon and Gloucestershire** | 11 | 64 | 16 | 9.2 |
| **South East London** | 0 | 40 | 60 | 0 |
| **South Yorkshire and Bassetlaw** | 12 | 55 | 32 | 0 |
| **Surrey and Sussex** | 1.7 | 37 | 48 | 14 |
| **Thames Valley** | 15 | 61 | 21 | 3 |
| **Wessex** | 5 | 78 | 18 | 0 |
| **West Midlands** | 31 | 29 | 20 | 20 |
| **West Yorkshire and Harrogate** | 25 | 50 | 25 | 0 |
| **Northern Ireland** | 25 | 58 | 18 | 0 |
| **Scotland** | 8 | 42 | 50 | 0 |
| **Wales** | 15 | 75 | 7.5 | 2.5 |
| **Overall** | **13** | **58** | **22** | **7.5** |

**Supplementary Table 7: Skin Cancer MDT attendance by SCSNs**

| **Cancer Alliance / Devolved Nation** | **Regular MDT attendance (% trusts)** | **Regular MDT leadership (% trusts)** | **Leading MDT Regularly (number of trusts)** | **Leading MDT Sometimes (number of trusts)** | **Never leading MDT (number of trusts)** |
| --- | --- | --- | --- | --- | --- |
| **Cheshire and Merseyside** | 88 | 38 | 3 | 1 | 4 |
| **East Midlands** | 100 | 14 | 1 | 2 | 4 |
| **East of England – North** | 100 | 17 | 1 | 1 | 4 |
| **East of England – South** | 100 | 0 | 0 | 1 | 3 |
| **Greater Manchester** | 75 | 0 | 0 | 0 | 4 |
| **Humber, Coast and Vale** | 100 | 100 | 1 | 0 | 0 |
| **Kent and Medway** | 100 | 0 | 0 | 1 | 1 |
| **Lancashire and South** | 100 | 0 | 0 | 0 | 3 |
| **North Central London** | 100 | 56 | 5 | 1 | 3 |
| **North East London** | 100 | 67 | 2 | 1 | 0 |
| **Northern** | 86 | 50 | 4 | 1 | 3 |
| **Peninsula** | 100 | 29 | 2 | 2 | 3 |
| **RM Partners West London** | 100 | 40 | 2 | 0 | 3 |
| **Somerset, Wiltshire, Avon and Gloucestershire** | 100 | 50 | 3 | 1 | 2 |
| **South East London** | 100 | 33 | 1 | 0 | 2 |
| **South Yorkshire and Bassetlaw** | 100 | 0 | 0 | 0 | 2 |
| **Surrey and Sussex** | 100 | 33 | 2 | 2 | 2 |
| **Thames Valley** | 83 | 33 | 2 | 0 | 4 |
| **Wessex** | 100 | 33 | 2 | 2 | 2 |
| **West Midlands** | 80 | 20 | 1 | 2 | 2 |
| **West Yorkshire and Harrogate** | 100 | 0 | 0 | 0 | 2 |
| **Northern Ireland** | 100 | 0 | 0 | 0 | 4 |
| **Scotland** | 100 | 20 | 1 | 1 | 3 |
| **Wales** | 100 | 50 | 1 | 0 | 1 |
| **Overall** | **96** | **30** | **34** | **19** | **61** |

**Supplementary Table 8: Main method of patient contact by SCSNs**

| **Cancer Alliance / Devolved Nation** | **F2F/ telephone consultation split (%) Jan 2022** | **F2F / telephone consultation split (%) Jan 2021** | **F2F/telephone consultation split (%) Jan 2022 (predicted)** |
| --- | --- | --- | --- |
| **Cheshire and Merseyside** | 79/21 | 73/27 | 84/16 |
| **East Midlands** | 74/26 | 75/25 | 85/15 |
| **East of England – North** | 51/49 | 51/49 | 68/32 |
| **East of England – South** | 64/46 | 28/72 | 67/22 |
| **Greater Manchester** | 80/20 | 58/42 | 79/21 |
| **Humber, Coast and Vale** | 80/20 | 50/50 | 50/50 |
| **Kent and Medway** | 45/55 | 35/65 | 63/37 |
| **Lancashire and South** | 83/17 | 80/20 | 83/17 |
| **North Central London** | 60/40 | 53/47 | 73/27 |
| **North East London** | 52/48 | 52/48 | 90/10 |
| **Northern** | 48/52 | 54/46 | 75/25 |
| **Peninsula** | 66/34 | 53/47 | 81/19 |
| **RM Partners West London** | 71/29 | 71/29 | 83/17 |
| **Somerset, Wiltshire, Avon and Gloucestershire** | 80/20 | 51/49 | 68/32 |
| **South East London** | 62/38 | 37/63 | 43/57 |
| **South Yorkshire and Bassetlaw** | 58/42 | 60/40 | 75/25 |
| **Surrey and Sussex** | 82/18 | 43/57 | 40/60 |
| **Thames Valley** | 81/19 | 52/48 | 80/20 |
| **Wessex** | 76/24 | 59/41 | 64/36 |
| **West Midlands** | 52/48 | 28/72 | 40/60 |
| **West Yorkshire and Harrogate** | 75/25 | 40/60 | 55/45 |
| **Northern Ireland** | 75/25 | 38/62 | 56/44 |
| **Scotland** | 48/52 | 44/56 | 67/33 |
| **Wales** | 75/25 | 55/45 | 88/12 |
| **Overall** | **68/32** | **52/48** | **69/31** |

**Supplementary Table 9: SCSN description of their job role**

|  | **Number of votes** | **% votes** |
| --- | --- | --- |
| **Challenging** | 103 | 29% |
| **Rewarding** | 102 | 29% |
| **Exhausting** | 47 | 13% |
| **Exciting** | 41 | 12% |
| **Overwhelming** | 35 | 10% |
| **Onerous** | 12 | 3% |
| **Fun** | 7 | 2% |
| **Depression** | 3 | 0.8% |
| **Dull** | 2 | 0.6% |
| **Boring** | 2 | 0.6% |

**Figure S1**: **Percentage of trusts within each cancer alliance/devolved nation that had 1 or more established SCSN post**


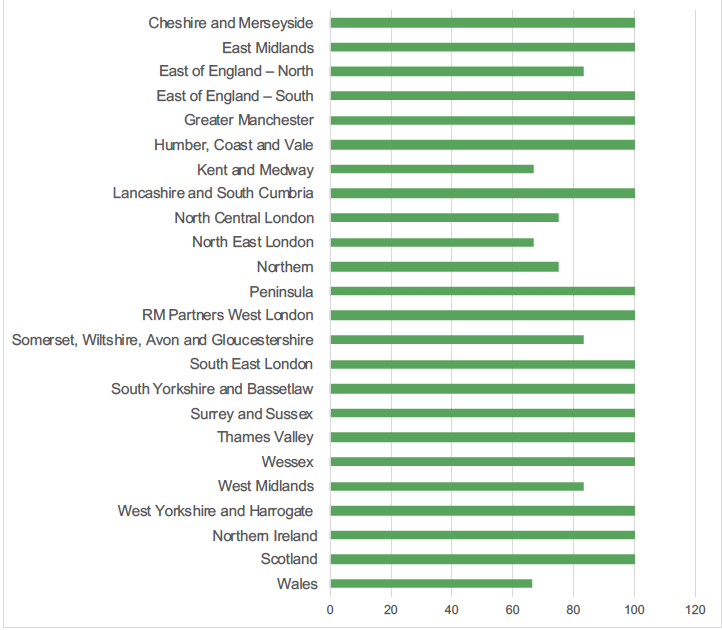


**Figure S2: SCSN WTEs across cancer alliances/devolved nations, ranges represent individual trusts.**


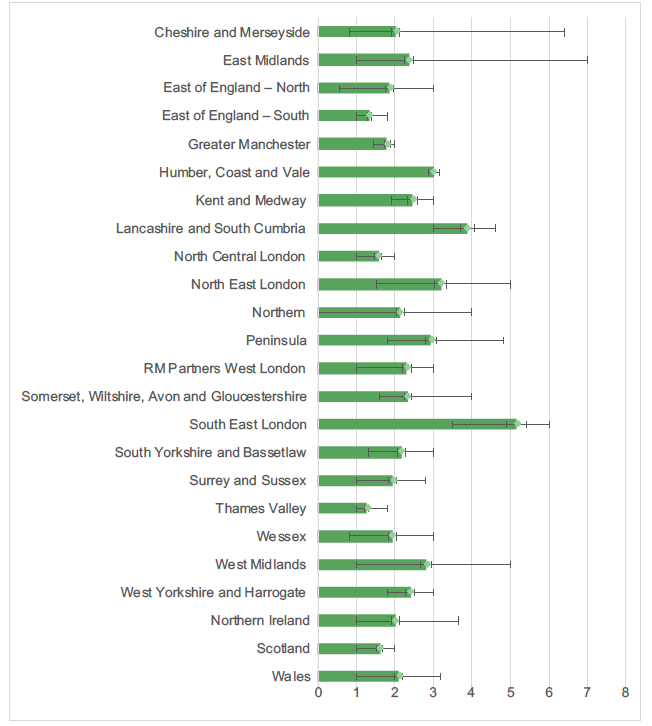


**Figure S3: Regional variation of skin cancer treatments available in trusts within each cancer alliance/devolved nation.** Colour code - green: available at all trusts; red: not available at any trusts; amber: available in some trusts

|  | **Surgery** | **Radiotherapy** | **Systemic therapy**  **(immunotherapy, targeted therapy)** | **Lymphoedema therapy** | **Photodynamic therapy (PDT)** | **Clinical trails**  **specifically for melanoma patients** | **Clinical trials for non-melanoma skin cancer**  **patients** |
| --- | --- | --- | --- | --- | --- | --- | --- |
| **Cheshire & Merseyside** |  |  |  |  |  |  |  |
| **East Midlands** |  |  |  |  |  |  |  |
| **East of England – North** |  |  |  |  |  |  |  |
| **East of England – South** |  |  |  |  |  |  |  |
| **Greater Manchester** |  |  |  |  |  |  |  |
| **Humber, Coast & Vale** |  |  |  |  |  |  |  |
| **Kent and Medway** |  |  |  |  |  |  |  |
| **Lancashire & South Cumbria** |  |  |  |  |  |  |  |
| **North Central London** |  |  |  |  |  |  |  |
| **North East London** |  |  |  |  |  |  |  |
| **Northern** |  |  |  |  |  |  |  |
| **Peninsula** |  |  |  |  |  |  |  |
| **RM Partners West London** |  |  |  |  |  |  |  |
| **Somerset,**  **Wiltshire, Avon & Gloucestershire** |  |  |  |  |  |  |  |
| **South East London** |  |  |  |  |  |  |  |
| **South Yorkshire and Bassetlaw** |  |  |  |  |  |  |  |
| **Surrey & Sussex** |  |  |  |  |  |  |  |
| **Thames Valley** |  |  |  |  |  |  |  |
| **Wessex** |  |  |  |  |  |  |  |
| **West Midlands** |  |  |  |  |  |  |  |
| **West Yorkshire & Harrogate** |  |  |  |  |  |  |  |
| **Northern Ireland** |  |  |  |  |  |  |  |
| **Scotland** |  |  |  |  |  |  |  |
| **Wales** |  |  |  |  |  |  |  |

**Figure S4: Proportion of time spent by SCSNs providing support to patients receiving different skin cancer treatments**


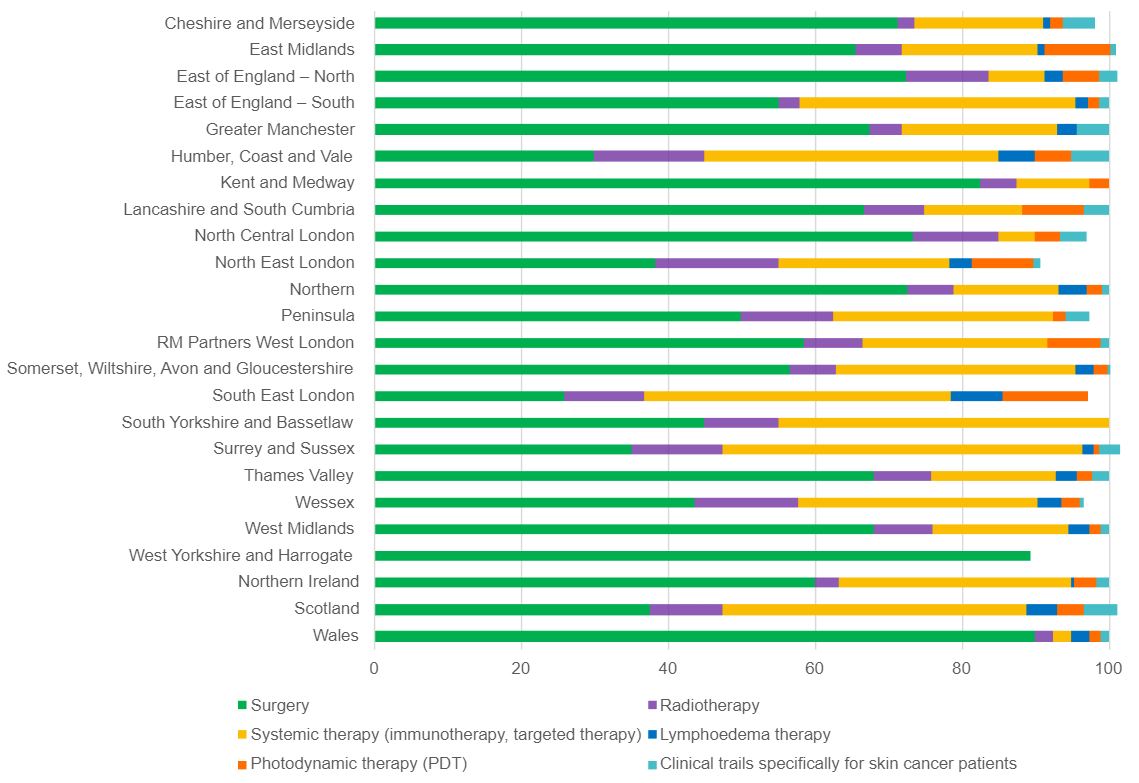


**Figure S5: Regional variation in type of work undertaken by SCSNs within each cancer alliance/devolved nation.** Colour code - green: undertaken at all trusts; red: not undertaken at any trusts; amber: undertaken in some trusts

|  | **Outpatients** | **Inpatients** | **Managing patients in the community** | **Liaison with community support teams** | **Managing patients in other secondary care trust(s)** | **Liaison with colleagues at other secondary care trust(s)** | **Prescribing** | **Independent assessment of patients** | **Ordering of tests such as blood tests, scan** |
| --- | --- | --- | --- | --- | --- | --- | --- | --- | --- |
| **Cheshire and Merseyside** |  |  |  |  |  |  |  |  |  |
| **East Midlands** |  |  |  |  |  |  |  |  |  |
| **East of England – North** |  |  |  |  |  |  |  |  |  |
| **East of England – South** |  |  |  |  |  |  |  |  |  |
| **Greater Manchester** |  |  |  |  |  |  |  |  |  |
| **Humber, Coast and Vale** |  |  |  |  |  |  |  |  |  |
| **Kent and Medway** |  |  |  |  |  |  |  |  |  |
| **Lancashire and South Cumbria** |  |  |  |  |  |  |  |  |  |
| **North Central London** |  |  |  |  |  |  |  |  |  |
| **North East London** |  |  |  |  |  |  |  |  |  |
| **Northern** |  |  |  |  |  |  |  |  |  |
| **Peninsula** |  |  |  |  |  |  |  |  |  |
| **RM Partners West London** |  |  |  |  |  |  |  |  |  |
| **Somerset, Wiltshire, Avon and Gloucestershire** |  |  |  |  |  |  |  |  |  |
| **South East London** |  |  |  |  |  |  |  |  |  |
| **South Yorkshire and Bassetlaw** |  |  |  |  |  |  |  |  |  |
| **Surrey and Sussex** |  |  |  |  |  |  |  |  |  |
| **Thames Valley** |  |  |  |  |  |  |  |  |  |
| **Wessex** |  |  |  |  |  |  |  |  |  |
| **West Midlands** |  |  |  |  |  |  |  |  |  |
| **West Yorkshire and Harrogate** |  |  |  |  |  |  |  |  |  |
| **Northern Ireland** |  |  |  |  |  |  |  |  |  |
| **Scotland** |  |  |  |  |  |  |  |  |  |
| **Wales** |  |  |  |  |  |  |  |  |  |

**Figure S6: Proportion of time spent by SCSNs working autonomously in different types of clinics**

**Figure S7: Skin Cancer MDT attendance by SCSNs**

**Figure S8: Case load means and trust ranges for each cancer alliance/devolved nation**


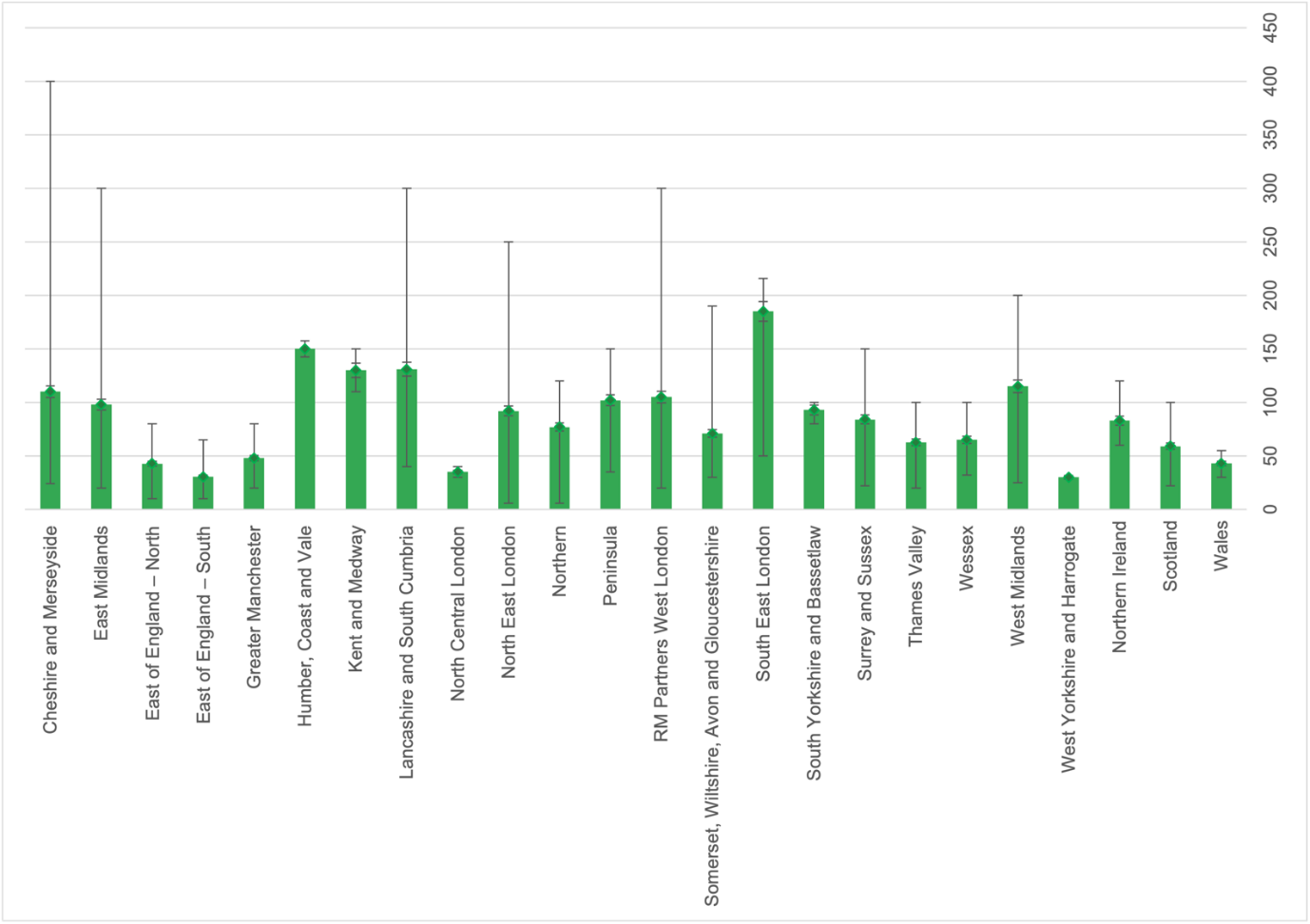


**Figure S9: Ratio of face-face and telephone/virtual patient contacts undertaken during January 2020 and 2021 and predicted for 2022; straight lines demonstrate averages across all regions**


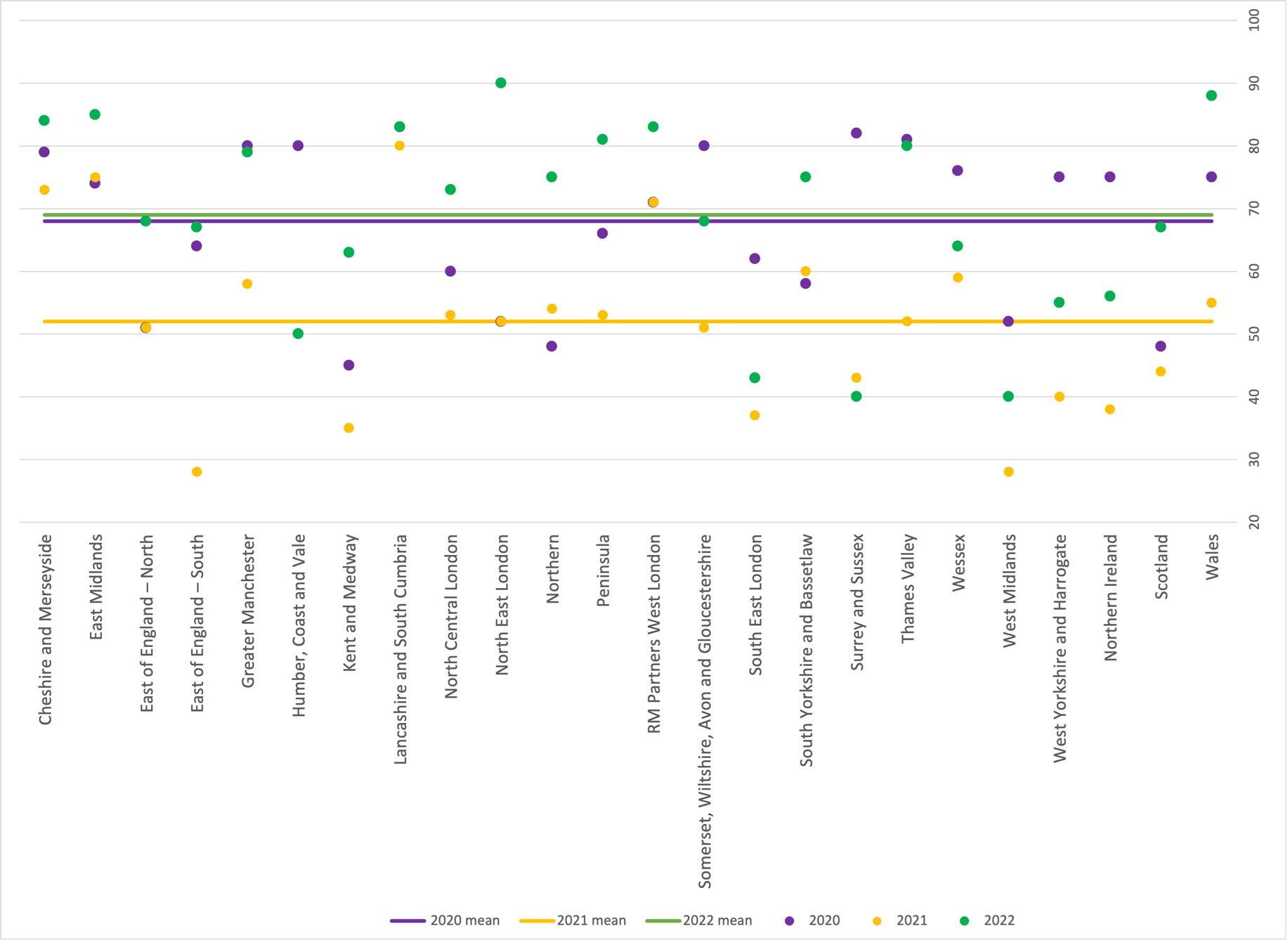

Supplement: Supplementary file 1 — Supplementary Material 1 [file 12912_2023_1374_MOESM1_ESM.docx]
